# *N*-[(*E*)-2-Chloro­benzyl­idene]-3-(4-methyl­benzyl­sulfan­yl)-5-(3,4,5-trimethoxy­phen­yl)-4*H*-1,2,4-triazol-4-amine

**DOI:** 10.1107/S1600536809009842

**Published:** 2009-03-25

**Authors:** Qian-Zhu Li, Bao-An Song, Song Yang, Yu-Guo Zheng, Qing-Qing Guo

**Affiliations:** a Center for Research and Development of Fine Chemicals, Guizhou University, Key Laboratory of Green Pesticide and Agricultural Bioengineering, Ministry of Education, Guiyang 550025, People’s Republic of China; b Department of Chemistry, Bijie University, Bijie 551700, People’s Republic of China

## Abstract

In the title compound, C_26_H_25_ClN_4_O_3_S, the acyclic imine group exhibits an *E* configuration. The triazole ring is oriented at dihedral angles of 53.84 (2), 70.77 (1) and 32.59 (3)° with respect to the benzene rings of the 2-chloro­benzyl­idene, 4-methyl­benzyl­sulfanyl and 3,4,5-trimethoxy­phenyl groups, respectively. The crystal packing is stabilized by weak inter­molecular C—H⋯N, C—H⋯S and C—H⋯π inter­actions.

## Related literature

For more information on 1,2,4-triazoles, see: He *et al.* (2006 ▶); Kritsanida *et al.* (2002 ▶); Demirbas *et al.* (2002 ▶); Chattopadhyay & Ghosh (1987 ▶, 1989 ▶).
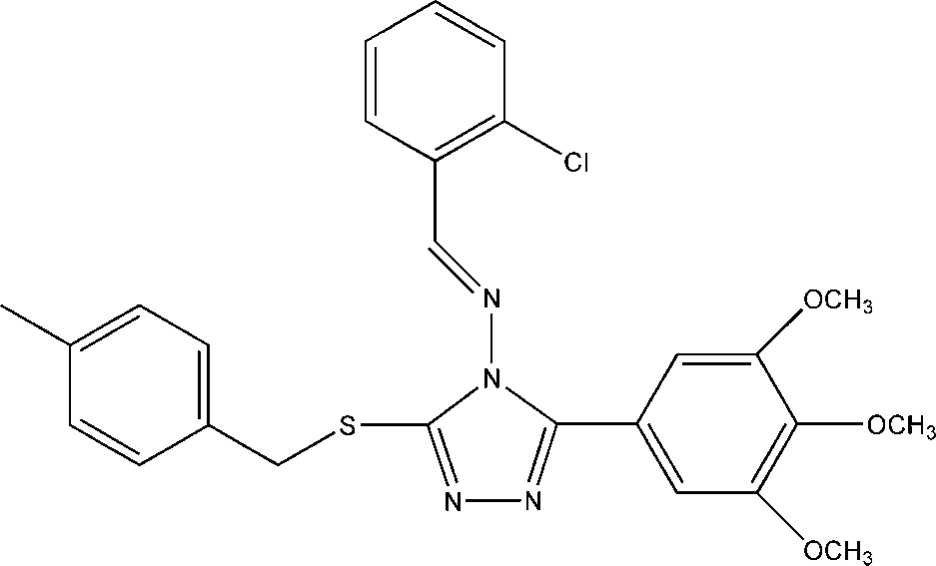


## Experimental

### 

#### Crystal data

C_26_H_25_ClN_4_O_3_S
*M* *_r_* = 509.01
Monoclinic, 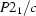

*a* = 11.283 (4) Å
*b* = 7.414 (2) Å
*c* = 31.087 (10) Å
β = 100.961 (14)°
*V* = 2553.1 (14) Å^3^
*Z* = 4
Mo *K*α radiation
μ = 0.27 mm^−1^
*T* = 293 K
0.32 × 0.26 × 0.22 mm

#### Data collection

Bruker APEXII CCD diffractometer
Absorption correction: multi-scan (*SADABS*; Sheldrick, 1996 ▶) *T*_min_ = 0.932, *T*_max_ = 0.956
26288 measured reflections
4590 independent reflections
3809 reflections with *I* > 2σ(*I*)
*R*_int_ = 0.028

#### Refinement

*R*[*F* ^2^ > 2σ(*F* ^2^)] = 0.051
*wR*(*F* ^2^) = 0.141
*S* = 1.03
4590 reflections
320 parameters
H-atom parameters constrained
Δρ_max_ = 0.50 e Å^−3^
Δρ_min_ = −0.65 e Å^−3^

### 

Data collection: *APEX2* (Bruker, 2004 ▶); cell refinement: *SAINT* (Bruker, 2004 ▶); data reduction: *SAINT*; program(s) used to solve structure: *SHELXS97* (Sheldrick, 2008 ▶); program(s) used to refine structure: *SHELXL97* (Sheldrick, 2008 ▶); molecular graphics: *DIAMOND* (Brandenburg, 2001 ▶); software used to prepare material for publication: *SHELXTL* (Sheldrick, 2008 ▶).

## Supplementary Material

Crystal structure: contains datablocks I, global. DOI: 10.1107/S1600536809009842/gk2192sup1.cif

Structure factors: contains datablocks I. DOI: 10.1107/S1600536809009842/gk2192Isup2.hkl

Additional supplementary materials:  crystallographic information; 3D view; checkCIF report

## Figures and Tables

**Table 1:** Hydrogen-bond geometry (Å, °)

*D*—H⋯*A*	*D*—H	H⋯*A*	*D*⋯*A*	*D*—H⋯*A*
C19—H19⋯N4	0.93	2.52	3.000 (3)	112
C10—H10⋯S1	0.93	2.81	3.184 (3)	105
C6—H6⋯N1^i^	0.93	2.61	3.409 (3)	144
C8—H8*B*⋯*Cg*2^ii^	0.97	2.70	3.427 (2)	133
C24—H24*B*⋯*Cg*1^iii^	0.96	2.94	3.588 (2)	125

Symmetry codes: (i) 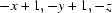; (ii) 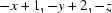; (iii) 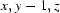. *Cg*1 is the centroid of the C9,C17,N1–N3 ring and *Cg*2 is the centroid of the C2–C7 ring.

**Fig. 1.**
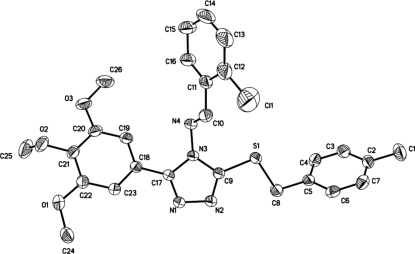
The structure of the title compound, showing the atomic numbering scheme. Non-hydrogen atoms are shown with 30% probability displacement ellipsoids.Hydrogen atoms have been omitted for clarity.

**Fig. 2.**
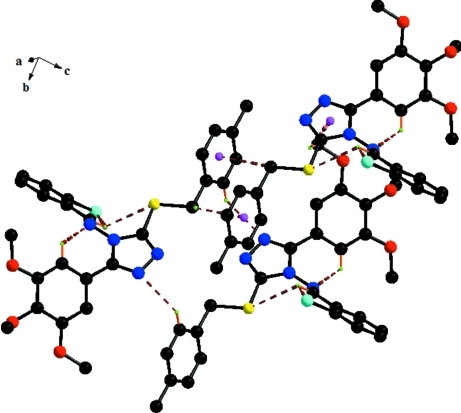
A packing view of the title compound. The interιntra molecluar C—H···N, C—H···S and C—H···π interactions are shown as dashed lines.

**Fig. 3.**
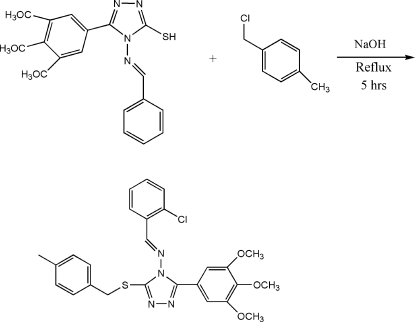
The synthesis procedure of the title compound.

## supplementary crystallographic information

### Comment

The design and synthesis of new 1,2,4-triazole derivatives is an important research field, since these species not only can be used to build polymetallic complexes (He *et al.*, 2006), but also show biological activity (Demirbas *et al., *2002; Kritsanida *et al., *2002). The biological activity is most probably due to the presence of the –N–C–S unit (Chattopadhyay & Ghosh, 1987, 1989). We are interested in the synthesis and biological activities of 1,2,4-triazole derivatives and report herein the synthesis and crystal structure of the title compound.

As illustrated in Figure 1, the 2-chlorobenzylidene, 4-methylbenzylsulfanyl, 3,4,5-trimethoxyphenyl and 1,2,4-triazole fragments are not coplanar with each other. The triazole ring is oriented with respect to the phenyl rings of 2-chlorobenzylidene, 4-methylbenzylthio and 3,4,5-trimethoxyphenyl units at dihedral angles of 53.84 (2)°, 70.77 (1) ° and 32.59 (3) °, respectively. The molecular packing is consolidated through weak inter- and intramolecular C—H···N, C—H···S and C—H···π interactions. C—H···π interactions of methylene H atoms and methyl H atoms are established towards the π-systems of neighboring aromatic groups from 4-methylbenzylsulfanyl and 1,2,4-triazole units (Table 1, Fig. 2, *Cg*1 = ring(C9,C17,N1—N3); *Cg*2 = ring(C2—C7)). ;

### Experimental

A mixture of 1-chloromethyl-4-methylbenzene (1.40 g, 0.01 mol) and methanol (5 ml) was added dropwise to a stirred solution of (*E*)-4-(2-chlorobenzylideneamino)-5-(3,4,5-trimethoxyphenyl) -4*H*-1,2,4-triazole-3-thiol (4.05 g, 0.01 mol) and sodium hydroxide (0.40 g, 0.01 mol) in water (20 ml). The resulting mixture was stirred at room temperature for 5 h. The precipitate formed was filtered off and recrystallized from ethanol to give pure title compound, which was then dissolved in 30 ml ethanol, and single crystals of the title compound were obtained after several days.

### Refinement

H atoms were placed in calculated positions and were treated as riding on the parent C atoms with C—H = 0.93 - 0.97 Å, and with *U*_iso_(H) = *xU*_eq_(C), where *x *= 1.2 for CH_2 _and CH groups and *x* = 1.5 for CH_3 _group.

### Crystal data

**Table:**

C_26_H_25_ClN_4_O_3_S	*F*(000) = 1064
*M**_r_* = 509.01	*D*_x_ = 1.324 Mg m^−^^3^
Monoclinic, *P*2_1_/*c*	Mo *K*α radiation, λ = 0.71073 Å
Hall symbol: -P 2ybc	Cell parameters from 2895 reflections
*a* = 11.283 (4) Å	θ = 2.4–27.9°
*b* = 7.414 (2) Å	µ = 0.27 mm^−^^1^
*c* = 31.087 (10) Å	*T* = 293 K
β = 100.961 (14)°	Block, colorless
*V* = 2553.1 (14) Å^3^	0.32 × 0.26 × 0.22 mm
*Z* = 4	

### Data collection

**Table:**

Bruker APEXII CCD diffractometer	4590 independent reflections
Radiation source: fine-focus sealed tube	3809 reflections with *I* > 2σ(*I*)
graphite	*R*_int_ = 0.028
φ and ω scans	θ_max_ = 25.2°, θ_min_ = 1.3°
Absorption correction: multi-scan (*SADABS*; Sheldrick, 1996)	*h* = −13→13
*T*_min_ = 0.932, *T*_max_ = 0.956	*k* = −8→8
26288 measured reflections	*l* = −36→37

### Refinement

**Table:**

Refinement on *F*^2^	Primary atom site location: structure-invariant direct methods
Least-squares matrix: full	Secondary atom site location: difference Fourier map
*R*[*F*^2^ > 2σ(*F*^2^)] = 0.051	Hydrogen site location: inferred from neighbouring sites
*wR*(*F*^2^) = 0.141	H-atom parameters constrained
*S* = 1.03	*w* = 1/[σ^2^(*F*_o_^2^) + (0.0627*P*)^2^ + 1.5591*P*] where *P* = (*F*_o_^2^ + 2*F*_c_^2^)/3
4590 reflections	(Δ/σ)_max_ < 0.001
320 parameters	Δρ_max_ = 0.50 e Å^−^^3^
0 restraints	Δρ_min_ = −0.65 e Å^−^^3^

### Special details

**Table:**

Geometry. All e.s.d.'s (except the e.s.d. in the dihedral angle between two l.s. planes) are estimated using the full covariance matrix. The cell e.s.d.'s are taken into account individually in the estimation of e.s.d.'s in distances, angles and torsion angles; correlations between e.s.d.'s in cell parameters are only used when they are defined by crystal symmetry. An approximate (isotropic) treatment of cell e.s.d.'s is used for estimating e.s.d.'s involving l.s. planes.
Refinement. Refinement of *F*^2^ against ALL reflections. The weighted *R*-factor *wR* and goodness of fit *S* are based on *F*^2^, conventional *R*-factors *R* are based on *F*, with *F* set to zero for negative *F*^2^. The threshold expression of *F*^2^ > σ(*F*^2^) is used only for calculating *R*-factors(gt) *etc*. and is not relevant to the choice of reflections for refinement. *R*-factors based on *F*^2^ are statistically about twice as large as those based on *F*, and *R*- factors based on ALL data will be even larger.

### Fractional atomic coordinates and isotropic or equivalent isotropic displacement parameters (Å^2^)

**Table:**

	*x*	*y*	*z*	*U*_iso_*/*U*_eq_	
Cl1	0.73726 (9)	0.3490 (3)	0.20121 (5)	0.1603 (7)	
S1	0.49049 (6)	0.68103 (9)	0.095977 (19)	0.0586 (2)	
O1	0.0266 (2)	−0.2541 (3)	0.08517 (7)	0.0766 (6)	
O2	−0.03565 (19)	−0.1835 (3)	0.16218 (6)	0.0837 (7)	
O3	0.01561 (19)	0.1311 (4)	0.20215 (6)	0.0879 (7)	
N1	0.23855 (18)	0.3418 (3)	0.04765 (6)	0.0526 (5)	
N2	0.32532 (18)	0.4699 (3)	0.04284 (6)	0.0540 (5)	
N3	0.32908 (16)	0.4276 (2)	0.11267 (5)	0.0454 (4)	
N4	0.35963 (19)	0.4375 (3)	0.15859 (6)	0.0556 (5)	
C1	0.8350 (3)	1.3689 (5)	0.06633 (12)	0.0904 (10)	
H1A	0.9129	1.3283	0.0623	0.136*	
H1B	0.8412	1.4179	0.0953	0.136*	
H1C	0.8059	1.4603	0.0451	0.136*	
C2	0.7483 (2)	1.2123 (4)	0.06056 (8)	0.0575 (6)	
C3	0.6369 (3)	1.2265 (4)	0.07290 (9)	0.0628 (7)	
H3	0.6165	1.3333	0.0854	0.075*	
C4	0.5560 (2)	1.0857 (4)	0.06705 (8)	0.0601 (6)	
H4	0.4817	1.0987	0.0755	0.072*	
C5	0.5842 (2)	0.9248 (3)	0.04874 (7)	0.0507 (5)	
C6	0.6947 (2)	0.9099 (3)	0.03637 (8)	0.0576 (6)	
H6	0.7157	0.8031	0.0240	0.069*	
C7	0.7742 (2)	1.0521 (4)	0.04224 (8)	0.0622 (7)	
H7	0.8481	1.0394	0.0335	0.075*	
C8	0.4967 (3)	0.7699 (4)	0.04208 (8)	0.0670 (7)	
H8A	0.5239	0.6775	0.0241	0.080*	
H8B	0.4175	0.8109	0.0276	0.080*	
C9	0.3782 (2)	0.5188 (3)	0.08193 (7)	0.0474 (5)	
C10	0.4692 (2)	0.4112 (4)	0.17385 (8)	0.0602 (6)	
H10	0.5216	0.3890	0.1547	0.072*	
C11	0.5165 (3)	0.4147 (4)	0.22074 (8)	0.0654 (7)	
C12	0.6388 (3)	0.3897 (5)	0.23688 (11)	0.0956 (12)	
C13	0.6842 (4)	0.3889 (7)	0.28080 (14)	0.1259 (18)	
H13	0.7664	0.3714	0.2910	0.151*	
C14	0.6090 (5)	0.4138 (6)	0.30980 (13)	0.1222 (18)	
H14	0.6403	0.4145	0.3397	0.147*	
C15	0.4867 (4)	0.4380 (5)	0.29504 (10)	0.1005 (12)	
H15	0.4353	0.4536	0.3148	0.121*	
C16	0.4418 (3)	0.4389 (4)	0.25060 (9)	0.0777 (8)	
H16	0.3596	0.4562	0.2406	0.093*	
C17	0.24118 (19)	0.3193 (3)	0.08948 (7)	0.0441 (5)	
C18	0.16596 (19)	0.1926 (3)	0.10855 (7)	0.0467 (5)	
C19	0.1265 (2)	0.2324 (4)	0.14714 (7)	0.0555 (6)	
H19	0.1468	0.3417	0.1613	0.067*	
C20	0.0568 (2)	0.1077 (4)	0.16425 (7)	0.0610 (7)	
C21	0.0254 (2)	−0.0554 (4)	0.14312 (8)	0.0602 (7)	
C22	0.0629 (2)	−0.0922 (3)	0.10397 (8)	0.0549 (6)	
C23	0.1337 (2)	0.0316 (3)	0.08673 (7)	0.0496 (5)	
H23	0.1594	0.0065	0.0607	0.060*	
C24	0.0497 (3)	−0.2881 (4)	0.04262 (11)	0.0803 (9)	
H24A	0.0168	−0.4034	0.0326	0.120*	
H24B	0.1353	−0.2882	0.0436	0.120*	
H24C	0.0127	−0.1957	0.0229	0.120*	
C25	−0.1619 (3)	−0.1711 (6)	0.15166 (12)	0.1129 (15)	
H52A	−0.1969	−0.2613	0.1675	0.169*	
H52B	−0.1884	−0.1897	0.1208	0.169*	
H52C	−0.1868	−0.0537	0.1595	0.169*	
C26	0.0534 (3)	0.2903 (6)	0.22675 (11)	0.1093 (15)	
H51A	0.0185	0.2923	0.2526	0.164*	
H51B	0.0274	0.3946	0.2092	0.164*	
H51C	0.1399	0.2910	0.2350	0.164*	

### Atomic displacement parameters (Å^2^)

**Table:**

	*U*^11^	*U*^22^	*U*^33^	*U*^12^	*U*^13^	*U*^23^
Cl1	0.0661 (6)	0.2676 (19)	0.1397 (10)	−0.0097 (8)	0.0008 (6)	0.0368 (11)
S1	0.0758 (4)	0.0572 (4)	0.0401 (3)	−0.0246 (3)	0.0041 (3)	0.0012 (3)
O1	0.0976 (15)	0.0598 (11)	0.0769 (13)	−0.0293 (11)	0.0278 (11)	−0.0043 (10)
O2	0.0840 (14)	0.1055 (17)	0.0620 (12)	−0.0282 (12)	0.0149 (10)	0.0257 (11)
O3	0.0803 (13)	0.142 (2)	0.0465 (10)	−0.0296 (13)	0.0240 (9)	−0.0154 (12)
N1	0.0573 (11)	0.0580 (12)	0.0409 (10)	−0.0129 (9)	0.0057 (8)	−0.0049 (9)
N2	0.0634 (12)	0.0568 (12)	0.0407 (10)	−0.0147 (10)	0.0074 (9)	−0.0014 (9)
N3	0.0525 (10)	0.0485 (10)	0.0333 (9)	−0.0078 (9)	0.0034 (7)	−0.0011 (8)
N4	0.0626 (13)	0.0667 (13)	0.0347 (9)	−0.0152 (10)	0.0018 (9)	−0.0041 (9)
C1	0.084 (2)	0.084 (2)	0.095 (2)	−0.0351 (18)	−0.0041 (18)	0.0141 (18)
C2	0.0582 (14)	0.0602 (15)	0.0507 (13)	−0.0121 (12)	0.0017 (11)	0.0112 (11)
C3	0.0749 (17)	0.0486 (14)	0.0661 (16)	−0.0038 (13)	0.0164 (13)	−0.0054 (12)
C4	0.0566 (14)	0.0632 (16)	0.0640 (15)	−0.0022 (12)	0.0202 (12)	0.0022 (13)
C5	0.0633 (14)	0.0513 (13)	0.0354 (11)	−0.0098 (11)	0.0039 (10)	0.0055 (10)
C6	0.0771 (17)	0.0533 (14)	0.0443 (13)	0.0041 (13)	0.0158 (11)	0.0028 (11)
C7	0.0558 (14)	0.0719 (18)	0.0606 (15)	0.0029 (13)	0.0151 (12)	0.0162 (13)
C8	0.0883 (19)	0.0647 (16)	0.0440 (13)	−0.0246 (15)	0.0027 (12)	0.0076 (12)
C9	0.0564 (13)	0.0434 (12)	0.0413 (12)	−0.0061 (10)	0.0068 (10)	0.0009 (9)
C10	0.0646 (16)	0.0654 (16)	0.0478 (13)	−0.0070 (13)	0.0037 (12)	0.0094 (12)
C11	0.0772 (18)	0.0624 (16)	0.0489 (14)	−0.0212 (14)	−0.0072 (13)	0.0105 (12)
C12	0.083 (2)	0.114 (3)	0.077 (2)	−0.037 (2)	−0.0177 (17)	0.0282 (19)
C13	0.117 (3)	0.147 (4)	0.088 (3)	−0.060 (3)	−0.047 (3)	0.040 (3)
C14	0.175 (5)	0.108 (3)	0.059 (2)	−0.059 (3)	−0.042 (3)	0.017 (2)
C15	0.156 (4)	0.094 (3)	0.0457 (16)	−0.030 (2)	0.0033 (19)	0.0046 (16)
C16	0.106 (2)	0.0747 (19)	0.0475 (14)	−0.0166 (17)	0.0013 (15)	0.0015 (14)
C17	0.0449 (11)	0.0462 (12)	0.0396 (11)	−0.0032 (9)	0.0039 (9)	−0.0044 (9)
C18	0.0419 (11)	0.0558 (13)	0.0403 (11)	−0.0035 (10)	0.0024 (9)	0.0001 (10)
C19	0.0525 (13)	0.0696 (16)	0.0436 (12)	−0.0080 (12)	0.0066 (10)	−0.0088 (11)
C20	0.0521 (13)	0.094 (2)	0.0372 (12)	−0.0081 (13)	0.0082 (10)	0.0009 (13)
C21	0.0555 (14)	0.0777 (18)	0.0460 (13)	−0.0160 (13)	0.0058 (10)	0.0113 (12)
C22	0.0547 (13)	0.0578 (14)	0.0505 (13)	−0.0087 (11)	0.0058 (11)	0.0044 (11)
C23	0.0512 (12)	0.0531 (13)	0.0443 (12)	−0.0063 (11)	0.0086 (10)	−0.0011 (10)
C24	0.093 (2)	0.0593 (17)	0.097 (2)	−0.0211 (16)	0.0402 (18)	−0.0241 (16)
C25	0.081 (2)	0.165 (4)	0.089 (2)	−0.055 (2)	0.0068 (18)	0.040 (2)
C26	0.097 (2)	0.172 (4)	0.067 (2)	−0.037 (3)	0.0355 (18)	−0.048 (2)

### Geometric parameters (Å, °)

**Table:**

Cl1—C12	1.738 (4)	C8—H8B	0.9700
S1—C9	1.741 (2)	C10—C11	1.454 (3)
S1—C8	1.814 (2)	C10—H10	0.9300
O1—C22	1.363 (3)	C11—C16	1.378 (4)
O1—C24	1.419 (3)	C11—C12	1.389 (4)
O2—C21	1.372 (3)	C12—C13	1.364 (5)
O2—C25	1.403 (4)	C13—C14	1.363 (7)
O3—C20	1.357 (3)	C13—H13	0.9300
O3—C26	1.427 (4)	C14—C15	1.380 (6)
N1—C17	1.306 (3)	C14—H14	0.9300
N1—N2	1.392 (3)	C15—C16	1.378 (4)
N2—C9	1.299 (3)	C15—H15	0.9300
N3—C17	1.370 (3)	C16—H16	0.9300
N3—C9	1.371 (3)	C17—C18	1.465 (3)
N3—N4	1.405 (2)	C18—C23	1.387 (3)
N4—C10	1.252 (3)	C18—C19	1.388 (3)
C1—C2	1.507 (4)	C19—C20	1.384 (4)
C1—H1A	0.9600	C19—H19	0.9300
C1—H1B	0.9600	C20—C21	1.390 (4)
C1—H1C	0.9600	C21—C22	1.390 (4)
C2—C7	1.373 (4)	C22—C23	1.389 (3)
C2—C3	1.385 (4)	C23—H23	0.9300
C3—C4	1.376 (4)	C24—H24A	0.9600
C3—H3	0.9300	C24—H24B	0.9600
C4—C5	1.385 (4)	C24—H24C	0.9600
C4—H4	0.9300	C25—H52A	0.9600
C5—C6	1.378 (4)	C25—H52B	0.9600
C5—C8	1.502 (3)	C25—H52C	0.9600
C6—C7	1.373 (4)	C26—H51A	0.9600
C6—H6	0.9300	C26—H51B	0.9600
C7—H7	0.9300	C26—H51C	0.9600
C8—H8A	0.9700		
			
C9—S1—C8	99.98 (11)	C14—C13—C12	120.0 (4)
C22—O1—C24	117.6 (2)	C14—C13—H13	120.0
C21—O2—C25	115.1 (2)	C12—C13—H13	120.0
C20—O3—C26	117.0 (2)	C13—C14—C15	120.4 (3)
C17—N1—N2	108.12 (17)	C13—C14—H14	119.8
C9—N2—N1	107.29 (18)	C15—C14—H14	119.8
C17—N3—C9	105.72 (17)	C16—C15—C14	119.2 (4)
C17—N3—N4	125.33 (18)	C16—C15—H15	120.4
C9—N3—N4	128.95 (18)	C14—C15—H15	120.4
C10—N4—N3	114.2 (2)	C15—C16—C11	121.3 (4)
C2—C1—H1A	109.5	C15—C16—H16	119.4
C2—C1—H1B	109.5	C11—C16—H16	119.4
H1A—C1—H1B	109.5	N1—C17—N3	109.03 (19)
C2—C1—H1C	109.5	N1—C17—C18	125.44 (19)
H1A—C1—H1C	109.5	N3—C17—C18	125.48 (19)
H1B—C1—H1C	109.5	C23—C18—C19	120.5 (2)
C7—C2—C3	117.2 (2)	C23—C18—C17	118.2 (2)
C7—C2—C1	122.1 (3)	C19—C18—C17	121.3 (2)
C3—C2—C1	120.7 (3)	C20—C19—C18	119.3 (2)
C4—C3—C2	121.3 (2)	C20—C19—H19	120.4
C4—C3—H3	119.3	C18—C19—H19	120.4
C2—C3—H3	119.3	O3—C20—C19	124.2 (3)
C3—C4—C5	120.6 (2)	O3—C20—C21	115.0 (2)
C3—C4—H4	119.7	C19—C20—C21	120.8 (2)
C5—C4—H4	119.7	O2—C21—C20	120.1 (2)
C6—C5—C4	118.3 (2)	O2—C21—C22	120.3 (3)
C6—C5—C8	120.4 (2)	C20—C21—C22	119.4 (2)
C4—C5—C8	121.3 (2)	O1—C22—C23	124.3 (2)
C7—C6—C5	120.3 (2)	O1—C22—C21	115.5 (2)
C7—C6—H6	119.9	C23—C22—C21	120.1 (2)
C5—C6—H6	119.9	C18—C23—C22	119.8 (2)
C2—C7—C6	122.3 (2)	C18—C23—H23	120.1
C2—C7—H7	118.9	C22—C23—H23	120.1
C6—C7—H7	118.9	O1—C24—H24A	109.5
C5—C8—S1	106.86 (16)	O1—C24—H24B	109.5
C5—C8—H8A	110.3	H24A—C24—H24B	109.5
S1—C8—H8A	110.3	O1—C24—H24C	109.5
C5—C8—H8B	110.3	H24A—C24—H24C	109.5
S1—C8—H8B	110.3	H24B—C24—H24C	109.5
H8A—C8—H8B	108.6	O2—C25—H52A	109.5
N2—C9—N3	109.84 (19)	O2—C25—H52B	109.5
N2—C9—S1	127.60 (17)	H52A—C25—H52B	109.5
N3—C9—S1	122.51 (16)	O2—C25—H52C	109.5
N4—C10—C11	121.6 (3)	H52A—C25—H52C	109.5
N4—C10—H10	119.2	H52B—C25—H52C	109.5
C11—C10—H10	119.2	O3—C26—H51A	109.5
C16—C11—C12	117.8 (3)	O3—C26—H51B	109.5
C16—C11—C10	121.5 (3)	H51A—C26—H51B	109.5
C12—C11—C10	120.6 (3)	O3—C26—H51C	109.5
C13—C12—C11	121.3 (4)	H51A—C26—H51C	109.5
C13—C12—Cl1	118.3 (3)	H51B—C26—H51C	109.5
C11—C12—Cl1	120.3 (3)		

### Hydrogen-bond geometry (Å, °)

**Table:**

*D*—H···*A*	*D*—H	H···*A*	*D*···*A*	*D*—H···*A*
C19—H19···N4	0.93	2.52	3.000 (3)	112
C10—H10···S1	0.93	2.81	3.184 (3)	105
C6—H6···N1^i^	0.93	2.61	3.409 (3)	144
C8—H8B···Cg2^ii^	0.97	2.70	3.427 (2)	133
C24—H24B···Cg1^iii^	0.96	2.94	3.588 (2)	125

Symmetry codes: (i) −*x*+1, −*y*+1, −*z*; (ii) −*x*+1, −*y*+2, −*z*; (iii) *x*, *y*−1, *z*.
